# Protective Effects of *Dendropanax morbifera* against Cisplatin-Induced Nephrotoxicity without Altering Chemotherapeutic Efficacy

**DOI:** 10.3390/antiox8080256

**Published:** 2019-07-30

**Authors:** Ji Su Kim, Kyeong Seok Kim, Ji Yeon Son, Hae Ri Kim, Jae Hyeon Park, Su Hyun Lee, Da Eun Lee, In Su Kim, Kwang Youl Lee, Byung Mu Lee, Jong Hwan Kwak, Hyung Sik Kim

**Affiliations:** 1 Division of Toxicology, School of Pharmacy, Sungkyunkwan University, Suwon 16419, Korea; 2 Division of Molecular Biology, College of Pharmacy & Research Institute of Drug Development, Chonnam National University, Gwangju 61186, Korea

**Keywords:** cisplatin, *Dendropanax morbifera*, renoprotective effect, antioxidants, chemotherapy, xenograft model

## Abstract

Use of the chemotherapeutic agent cisplatin (CDDP) in cancer patients is limited by the occurrence of acute kidney injury (AKI); however, no protective therapy is available. We aimed to investigate the renoprotective effects of *Dendropanax morbifera* water extract (DM) on CDDP-induced AKI. Male Sprague-Dawley rats (six animals/group) received: Vehicle (control); CDDP (6 mg/kg, intraperitoneally (i.p.); DM (25 mg/kg, oral); or DM + CDDP injection. CDDP treatment significantly increased blood urea nitrogen (BUN), serum creatinine (sCr), and pro-inflammatory cytokines (IL-6 and TNF-α), and severely damaged the kidney architecture. Urinary excretion of protein-based AKI biomarkers also increased in the CDDP-treated group. In contrast, DM ameliorated CDDP-induced AKI biomarkers. It markedly protected against CDDP-induced oxidative stress by increasing the activity of endogenous antioxidants and reducing the levels of pro-inflammatory cytokines (IL-6 and TNF-α). The protective effect of DM in the proximal tubules was evident upon histopathological examination. In a tumor xenograft model, administration of DM enhanced the chemotherapeutic activity of CDDP and exhibited renoprotective effects against CDDP-induced nephrotoxicity without altering chemotherapeutic efficacy. Our data demonstrate that DM may be an adjuvant therapy with CDDP in solid tumor patients to preserve renal function.

## 1. Introduction

Cisplatin (CDDP) is one of the most effective chemotherapeutic agents used widely for the treatment of malignant tumors in the testes, ovaries, cervix, lung, bladder, stomach, and many other organs [1]. While CDDP induces various side effects including ototoxicity, gastrotoxicity, myelosuppression, and allergic reactions [2,3], nephrotoxicity is one of the very serious side effects that has been observed in clinical practice. CDDP-induced nephrotoxicity can present with various types of symptoms including acute kidney injury (AKI), hypomagnesemia, renal tubular acidosis, hypocalcemia, and hyperuricemia [4,5]. However, the most serious aspect of CDDP-induced complication is acute kidney injury (AKI), which occurs in 20–30% of patients [6]. Clinical data have shown that approximately one-third of patients experience AKI after CDDP therapy, with reduced glomerular filtration rate, increased blood urea nitrogen (BUN) and serum creatinine (sCr), and imbalanced electrolytes [7,8]. CDDP-induced renal injury is generally characterized by tubular degeneration and necrosis localized to the S3 segment of the proximal tubules with loss of microvilli, alterations in the number and size of lysosomes, and mitochondrial vacuolation [9].

Natural products are known to have protective effects against CDDP-induced AKI by scavenging free radicals and modulating the antioxidant defense system [10]. In particular, dietary supplements are popular complementary products for the prevention and therapy of renal injury. *Dendropanax morbifera* (DM, *D. morbifera*) is traditionally used as a folk medicine for the treatment of various diseases including inflammation, migraines, menstrual pain, and wind dampness [11,12]. Previous research has demonstrated that the extracts of *D. morbifera* roots, leaves, and stems contain biologically active compounds, such as polyphenols and polyacetylene, that exhibit potent antioxidant, anti-inflammatory, anticancer, anticomplement, and antidiabetic properties [13,14,15]. In addition, the organic solvent extracts of *D. morbifera* have potent antioxidant effects against cancer, diabetes, hepatic fibrosis, and kidney toxicity [16,17,18]. However, the protective effects of the aquatic extract (DM) from leaves and stems of *D. morbifera* against CDDP-induced AKI is not clearly understood. Thus, the aim of the present study was to investigate the protective role of DM against CDDP-induced nephrotoxicity in an animal model. To the best of our knowledge, this investigation is the first report to examine the molecular mechanisms underlying the effects of DM on the prevention and strategy of CDDP chemotherapy.

## 2. Materials and Methods

### 2.1. Chemicals and Reagents

CDDP was purchased from Sigma-Aldrich (St. Louis, MO, USA) and was used to induce kidney injury. CDDP was dissolved in normal saline (0.9% sodium chloride). Primary antibodies against neutrophil gelatinase-associated lipocalin (NGAL), kidney injury molecule-1 (KIM-1), selenium binding protein-1 (SBP1), and Ki-67 were purchased from Abcam (Cambridge, MA, USA). Assay kits used to measure BUN and sCr were purchased from Abcam. Superoxide dismutase (SOD) and catalase assay kits were purchased from Cayman Chemical (Ann Arbor, MI, USA). TUNEL assay kits were purchased from Promega (Promega, Madison, WI, USA). All other chemicals used in this study were purchased from Sigma-Aldrich.

### 2.2. Plant Material and Preparation of Water Extracts from D. morbifera

Aerial parts of *D. morbifera* were harvested from a cultivation farm at Gwangyang-si, Jeollanam-do, Korea in October 2016. A voucher specimen was deposited in the specimen room, School of Pharmacy, Sungkyunkwan University (SKKU-Ph-16-031). Dried aerial parts of *D. morbifera* (100 g) were extracted with water at 95 °C (2 × 1 L, for 5 h), and the extracts were concentrated under reduced pressure to prepare a water extract of *D. morbifera* with the constant volume of 1 L.

### 2.3. HPLC Analysis of the Water Extract of D. morbifera 

**Figure 1:**
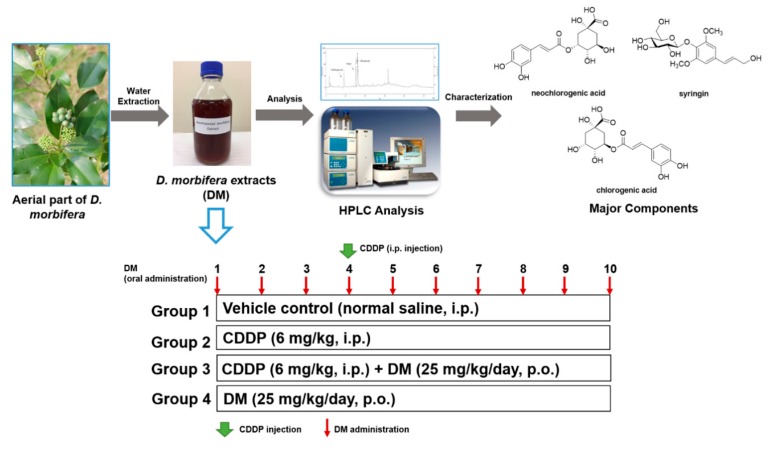
Preparation of *Dendropanax morbifera* water extract (DM) and experimental design of cisplatin (CDDP)-induced nephrotoxicity in rats. Rats were randomly divided into four groups: The control group, which received normal saline; the CDDP (6 mg/kg, single intraperitoneal (i.p.) injection) group; the CDDP (6 mg/kg, single i.p. injection) + DM (25 mg/kg/day) group; and the DM (25 mg/kg/day) group. The DM was administered orally for 10 days.

To identify the potential bioactive components of DM extract, the High Performance Liquid Chromatography (HPLC) analysis of DM extract were conducted using a reversed-phase (RP) C_18_ HPLC using gradient elution. HPLC analysis was performed using a Knauer HPLC system consisting of a Manager 5000, two Pump 1000 devices, a UV Detector 2500, and a Phenomenex Kinetex C18 column (150 × 4.6 mm, 5 µm). The mobile phases consisted of acetonitrile (A) and/or water with 0.3% phosphoric acid (B). The gradient profile used was 5% A (0–5 min, isocratic elution), 5–15% A (5–25 min, linear change), 15–30% A (25–35 min, linear change), 30% A (35–40 min, isocratic elution), 30–100% A (40–50 min, linear change), and 100% A (50–55 min, isocratic elution) at a flow rate of 1 mL/min. The column oven temperature and UV absorption were set at 30 °C and 254 nm wavelength, respectively. Standard working solutions for 3 major components were prepared by serial dilution with methanol/water (1:1) to yield concentrations of 500, 200, 100, and 50 μg/mL, respectively. Three major components were detected as major peaks at 254 nm and quantified using a regression equation for each compound (Figure 1).

### 2.4. Experimental Design

Male Sprague-Dawley rats (6 weeks old, 200 ± 5 g) were purchased from Charles River Laboratory Animal Resources (OrientBio, Sungnam, Korea). All animals were maintained in a specific-pathogen free (SPF) room with a 12 h light/dark cycle under automatically controlled temperature (23 ± 0.5 °C) and humidity (55 ± 2%) conditions. Tap water and rodent chow were provided ad libitum. All animals were acclimated for 1 week before experiments. The experimental protocol was approved for ethical procedures and scientific care by the Sungkyunkwan University Institutional Animal Care and Use Committee (SKKUIACUC2018-08-13-2).

CDDP (6 mg/kg) was dissolved in normal saline (0.9% sodium chloride) and intraperitoneally (i.p.) injected into rats once over 10 days to induce kidney injury. Then, the rats were divided into four groups (*n* = 6 per group), as shown in Figure 1. These groups included: Vehicle control (normal saline, i.p.), CDDP (6 mg/kg, i.p.), pretreatment with CDDP (6 mg/kg) 1 h prior to the administration of DM (25 mg/kg/kg), and DM alone (25 mg/kg, dissolved in deionized distilled water). At the end of the experiment, all animals were sacrificed after fasting for 24 h. The kidney was isolated for histological and other analyses. After the collection of blood samples at 60 min, serum was separated by centrifugation at 4000 rpm. Kidney and serum samples were immediately stored at −80 °C until analysis.

### 2.5. Urinalysis and Serum Biochemical Analysis

For urine collection, the animals were moved into a metabolic cage for 24 h before euthanizing. Total urine samples were collected over a 24 h period, and the exact urine volume was recorded. The urine samples were immediately centrifuged at 900× *g* for 10 min to remove impurities. Urine stored at −80 °C was slowly dissolved at 4 °C to use for analysis. The rats were fasted overnight and anesthetized by exposure to CO_2_. Blood was collected from the abdominal aorta and immediately centrifuged for 10 min at 1500× *g*. The serum samples were then immediately stored at −80 °C until analysis. Biochemical analysis of various parameters, including sCr and BUN, was performed using a Hitachi 912 Automatic Analyzer (Roche Diagnostics, Sandhofer, Mannheim, Germany).

### 2.6. Histopathological Examination

Sections of the left kidney were fixed in 10% neutral phosphate-buffered formalin (pH 7.4) and dehydrated through ascending grades of alcohol and embedded in paraffin. Tissues were sliced into approximately 5 µm thick sections and deparaffinized with xylene (3 × 7 min). The sections were rehydrated in a graded alcohol series, washed with deionized water, and stained with Haemotoxylin and Eosin (H&E) (Sigma-Aldrich, St. Louis, MO, USA) for 8 min. The slices were then washed under running tap water for 5 min and rinsed with 10 drops of 95% alcohol. After washing with alcohol, the sections were counterstained in eosin-phloxine solution for 1 min, dehydrated using 95% alcohol, with two changes of absolute alcohol for 5 min, and cleared in two changes of xylene for 5 min. Finally, the tissues were mounted with a xylene-based mounting medium. The histopathological findings were determined by examining the samples through a light microscope (Nanoscope Systems, Daejeon, Korea).

### 2.7. Determination of Antioxidant Enzyme Activity

Catalase content was measured following the manufacturer’s instructions for the assay kit (Abcam, Cambridge, MA, USA). Kidney tissues were washed with Phosphate-buffered saline (PBS), homogenized in ice-cold assay buffer, and centrifuged (10,000× *g*) for 15 min at 4 °C. The supernatant was transferred to a new sterile tube and stored at 4 °C. Catalase was measured using a UV spectrophotometer at 570 nm. Catalase activity was expressed as nmol/min/mL protein and quantitated using a standard curve. SOD content was measured using the manufacturer’s analytical kit (Cayman chemical, Ann Arbor, Michigan, USA). Kidney tissues were washed with PBS, homogenized in HEPES buffer (containing EGTA, mannitol, sucrose, pH 7.2) and centrifuged at 1500× *g* for 5 min at 4 °C. Each sample was added to the kidney tissue samples and the SOD content was measured at 440 nm. SOD was expressed as U/mL and quantified using a standard curve. SOD (U/mL) = [(Sample linearized rate (LR) – y− intercept/slope) × 0.23 mL/0.01 mL] × sample dilution.

### 2.8. Analysis of Inflammatory Cytokines

The effects of DM extracts on the levels of CDDP-induced inflammatory cytokines were measured by ELISA assay kits (Abcam). All cytokine-based assays were performed according to the manufacturer’s instructions. Key cytokines including TNF-α, IL-1β, IL-6, and IL-10 levels were assessed from standard plots.

### 2.9. Western Blot Analysis

Frozen urine was thawed approximately 1 h before analysis; thereafter, it was vortexed and centrifuged at 3000× *g* to filter out impurities. Frozen kidney samples were homogenized in PRO-PREPTM^TM^ protein extract solution (iNtRON, Seongnam, Korea) and centrifuged at 12,000× *g* for 10 min. Protein concentrations were measured using a protein assay kit (Bio-Rad, Hercules, CA, USA) according to the manufacturer’s instructions. Equal amounts of protein (10 μg) were loaded onto 6–15% SDS-PAGE gel. After electrophoresis, the gels were transferred to a polyvinylidene difluoride (PVDF) membrane (Millipore, Billerica, MA, USA). After transfer, the membranes were blocked at 25 °C for 1 h using 10 mM Tris-Cl, pH 7.6, 100 mM NaCl, and 0.5% Tween 20 (TNT buffer) containing 5% skim milk. Then, the membrane was incubated with primary antibodies against KIM-1 (1:500), SBP1 (1:500), and NGAL (1:500) at 4 °C for overnight and washed twice, for 1 min each time. Subsequently, membranes were incubated with anti-mouse IgG (1:20,000) or anti-rabbit IgG (1:20,000) horseradish peroxidase (HRP)-conjugate for 60 min at room temperature. Once incubated with the secondary antibody, membranes were washed once for 1 min and twice for 10 min with PBS-Tween 20. Finally, the blots were developed by using an enhanced chemiluminescence (ECL)-plus kit (Amersham Biosciences, Amersham, Buckinghamshire, UK).

### 2.10. Immunohistochemical Examination

Slide sections of kidney and tumor tissues were treated with xylene and ethanol and then boiled in sodium citrate buffer for 20 min. Tissue sections were treated with 5% H_2_O_2_ for 15 min to inactivate endogenous peroxidase. Kidney slides were reacted with KIM-1 (1:1000), SBP1 (1:1000), and NGAL (1:1000) antibodies. Slides were reacted with Ki-67 (1:1000) at 4 °C for 24 h. The secondary anti-rabbit IgG (VECTOR laboratories, Burlingame, CA, USA) was allowed to react at room temperature for 30 min. HRP-streptavidin reagent (VECTOR laboratories) was reacted at room temperature for 30 min. Following staining by DAB (Dako, Agilent, Santa Clara, CA, USA) and hematoxylin (Dako), the slides were fixed on a cover glass using a mounting solution after ethanol and xylene treatment. Slides were observed at 200× magnification by a confocal K1-fluo microscope (Nanoscope Systems, Daejeon, Korea). 

### 2.11. TUNEL Assay

Apoptosis was evaluated according to the TUNEL method using the DeadEnd™ Colorimetric TUNEL System (Promega, Madison, Wisconsin, USA). The kidney sections were deparaffinized with xylene and rehydrated through a graded series of ethanol and digested with proteinase K (20 µg/mL) for 20 min. The sections were washed twice in PBS and incubated with reaction buffer for 5 min. dUTP-digoxigenin was incorporated in the presence of working-strength terminal deoxynucleotide transferase (TdT) for 1 h at 37 °C in a humidified chamber. After the reaction was terminated using stop-wash buffer, the anti-digoxigenin conjugate was applied and the color was allowed to develop in 3,3′-diaminobenzidine hydrochloride (DAB) with hydrogen peroxide as the substrate for 30 min in the dark. The sections were washed in running buffer for 5 min and counterstained with haematoxylin for 5 min. Finally, slides were observed at 100× magnification using a K1-fluo microscope (Nanoscope Systems, Daejeon, Korea).

### 2.12. In Vivo Tumor Xenograft Model

Four-week-old male BALB/c nude mice weighing approximately 20 g (Japan SLC Inc., Hamamatsu, Shizuoka, Japan) were housed under controlled temperature conditions (22 ± 2 °C) and a 12 h light/dark cycle in laminar flow cabinets with filtered air. The mice were handled using aseptic procedures. The experimental procedure was approved by the Institutional Animal Care Committee of Sungkyunkwan University (SKKUIACUC2018-11-01-1). Mice were injected subcutaneously with HCT-116 cells (2 × 10^7^ cell/0.1 mL) in serum-free medium containing 50% Matrigel™ (BD Biosciences, Franklin Lakes, NJ, USA). Mice were divided into 4 groups (6 animals per group): (a) Treatment with 0.2 mL PBS vehicle; (b) CDDP treatment (4 mg/kg, i.p.); (c) administration of both DM (25 mg/kg/day, oral gavage) + CDDP (4 mg/kg, i.p.); and (d) administration of DM (25 mg/kg/day) by oral gavage. CDDP was injected once per week for 30 days. DM was administered daily for 30 days by oral gavage. Tumor volume (V) was calculated using the standard formula, V (mm^3^) = 0.52 (*ab*^2^), where *a* is the length and *b* is the width of the tumor. Body weight was recorded before drug administration and at the time of experiment termination. On day 31, the mice were euthanized by CO_2_ asphyxiation.

### 2.13. Statistical Analysis

Statistical analyses were performed using GraphPad Prism (version 5.0 for Windows) statistical software package (GraphPad Software, San Diego, CA, USA). The data are presented as the mean ± standard deviation (SD). One-way analysis of variance (ANOVA) and Bonferroni tests were used to determine the level of significance among the group. *p* < 0.05 was considered significantly different.

## 3. Results

### 3.1. Phytochemical Characterization of DM

**Figure 2:**
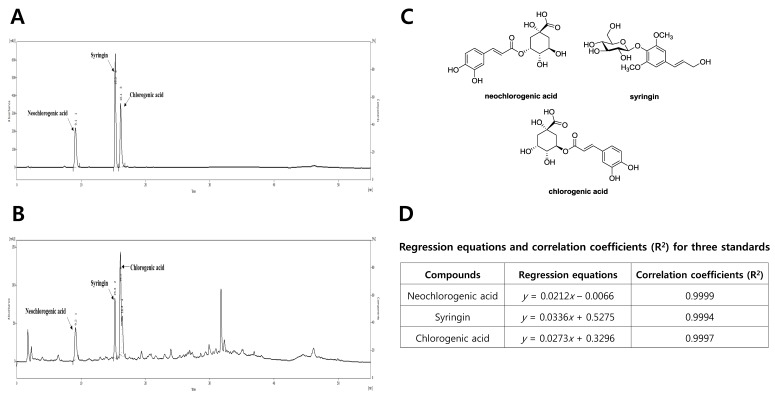
Phytochemical characterization of DM by HPLC analysis. (**A**) HPLC-UV chromatogram for DM; (**B**) HPLC-UV chromatogram for three standard compounds (neochlorogenic acid, syringin, and chlorogenic acid); (**C**) chemical structures of three major compounds in DM. (**D**) Regression equations and correlation coefficients (*R*^2^) for the three standard compounds. HPLC analysis was carried out via gradient elution on a Phenomenex Kinetex C18 column (150 × 4.6 mm, 5 µm). The flow rate, column oven temperature, and UV wavelength for detection were set at 1 mL/min, 30 °C, and 254 nm, respectively.

To characterize the DM, the major peaks were identified using reversed-phase HPLC analysis. Further, three major peaks were observed in the HPLC-UV chromatogram of DM (Figure 2A) and were identified as neochlorogenic acid, syringin, and chlorogenic acid by comparing their standard materials (Figure 2B,C). Using regression equations of the three compounds, the contents of neochlorogenic acid, syringin, and chlorogenic acid in the DM were calculated as 90.43, 33.24, and 125.23 μg/mL, respectively (Figure 2D).

### 3.2. Effects of DM on Body and Kidney Weight Changes in CDDP-Treated Rats

**Figure 3:**
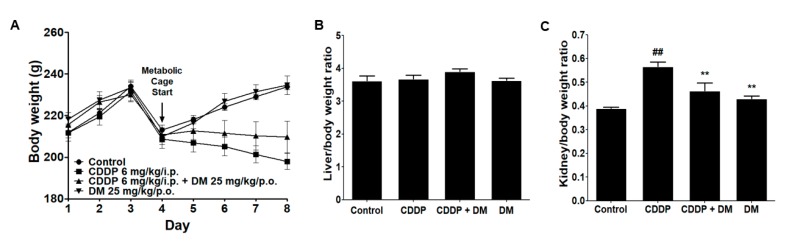
Effects of DM on changes in body, liver, and kidney weights in rats treated with CDDP. (**A**) Body weight changes in CDDP-treated rats. (**B**) Liver weight in CDDP-treated rats. (**C**) Kidney weight in CDDP-treated rats. All values represent the mean ± Standard Deviation (SD) of six rats per group. Statistical analyses were performed by one-way ANOVA followed by Tukey’s HSD (honest significant difference) post hoc test for multiple comparisons. ^##^ *p* < 0.01 compared to the control group; ** *p* < 0.01 compared to the CDDP-treated group.

Rats in the CDDP-treated group presented a significant decrease in body weight (Figure 3A). The liver weight did not change in any of the experiment groups (Figure 3B). However, kidney weights were considerably increased in the CDDP-treated group compared with the control group. In the DM group, the kidney weight was significantly reduced compared with that in the CDDP group (Figure 3C). Therefore, these results indicate that DM may protect against CDDP-induced kidney injury in rats.

### 3.3. Protective Effect of DM on CDDP-Induced AKI

**Figure 4:**
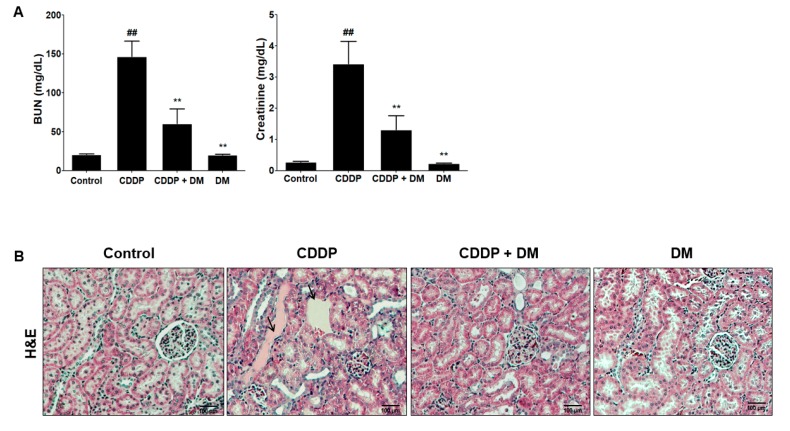
Effects of DM on CDDP-induced nephrotoxicity in rats. (**A**) Serum biochemical parameters were measured in CDDP-treated rats. Values are the mean ± SD of six rats per group. Statistical analyses were performed using one-way ANOVA followed by Tukey’s HSD post hoc test for multiple comparisons. ^##^ *p* < 0.01 compared to the control group; ** *p* < 0.01 compared to the CDDP-treated group. (**B**) H&E staining of representative kidney tissues from all experimental groups. Black arrows indicate renal tissue necrosis and infiltration in the proximal tubules. Magnification ×100.

As shown in Figure 4A, renal injury biomarkers (BUN and sCr levels) were significantly increased in the CDDP-treated group compared with the control group. These results indicate the presence of severe AKI in rats after CDDP (6 mg/kg, i.p.) injection. Administration with DM significantly ameliorated BUN and sCr levels in CDDP-treated rats. However, no changes in BUN and sCr were observed following DM administration alone. For histopathological examination, the proximal tubules and glomerular damages were visualized using H&E staining. In the control group, normal morphology in the glomeruli and the proximal tubules, as well as the vessels, were clearly shown in the kidneys of rats (Figure 4B). However, abnormal injuries such as hydropic swelling and hypertrophy in proximal tubules were observed in the kidneys of CDDP-treated rats. DM administration markedly protected CDDP-induced proximal tubular damage and disruption of renal architecture. Taken together, these data indicate that DM administration showed protective effects against CDDP-induced proximal tubular epithelial cell damage. In the rats administered DM alone, no changes in the structure or signs of renal damage were observed. The histological examination results were very similar to the observed serum biochemical parameters.

### 3.4. Effect of DM on the Urinary Excretion of AKI Biomarkers in CDDP-Treated Rats

**Figure 5:**
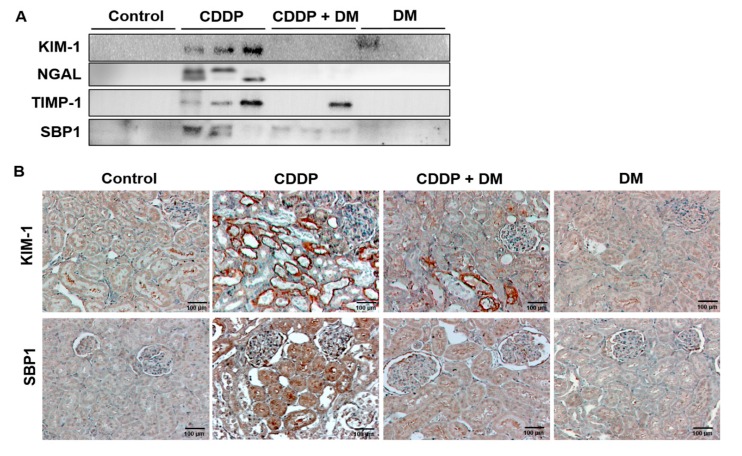
Effect of DM on acute kidney injury biomarkers in CDDP-treated rats. (**A**) Changes in the urinary excretion of KIM-1, NGAL, TIMP-1, and SBP1. The Western blot results represent three separate experiments. (**B**) Immunohistochemical staining of KIM-1 and SBP1 in the kidney of the rat. Magnification ×100.

The urinary excretion of AKI biomarkers was measured by Western blotting. As shown in Figure 5A, CDDP (6 mg/kg) treatment significantly increased the urinary excretion of KIM-1, NGAL, TIMP-1, and SBP1 compared with the controls. However, the administration of DM (25 mg/kg) markedly reduced the urinary excretion of these biomarkers in CDDP-treated rats. In immunochemical staining, KIM-1 and SBP1 levels were mainly expressed in the damaged proximal tubules of the kidney (Figure 5B). Similar to the changes in urinary biomarkers, KIM-1 and SBP1 were highly expressed in the kidney of CDDP-treated animals. However, DM administration markedly reduced the expression of KIM-1 and SBP1 in the kidneys of rats. Therefore, KIM-1 and SBP1 proteins were found to be significantly excreted in the urine according to the degree of kidney damage.

### 3.5. Effect of DM on Antioxidant Enzyme Activity and Pro-Inflammatory Cytokines in CDDP-Treated Rats

**Figure 6:**
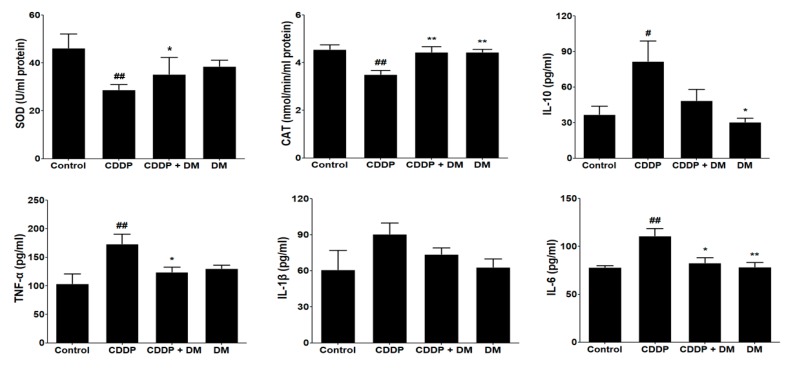
Effect of DM on antioxidant enzyme activities and pro-inflammatory cytokine levels in CDDP-treated rats. The activities of superoxide dismutase (SOD) and catalase were measured in the kidney of rats. Pro-inflammatory cytokines levels were measured in the serum of rats. Values are the mean ± SD of six rats per group. Statistical analyses were performed using one-way ANOVA followed by Tukey’s HSD post hoc test for multiple comparisons. ^#^ *p* < 0.05 and ^##^ *p* < 0.01 compared to the control group; * *p* < 0.05 and ** *p* < 0.01 compared to the CDDP-treated group.

The effects of DM on CDDP-induced oxidative stress were measured in the kidney. The CDDP-treated group presented significant reductions in SOD and catalase activities (Figure 6). However, administration of DM markedly elevated the antioxidant enzymes activity reduced in response to CDDP-induced oxidative stress. Previous studies indicated that *D*. *morbifera* extract was effective in attenuating the inflammatory response [19,20,21]. As demonstrated in Figure 6, levels of pro-inflammatory cytokines such as TNF-α, IL-1β, and IL-6 were significantly increased in the CDDP-treated group, with a 1.5-fold induction in the levels of these cytokines. However, administration of DM exerted a significant inhibitory effect on pro-inflammatory cytokines in CDDP-treated rats (Figure 6). IL-10 levels were significantly reduced in CDDP-treated rats by DM administration.

### 3.6. Effect of DM on Apoptosis in the Kidney of CDDP-Treated Rats

**Figure 7:**
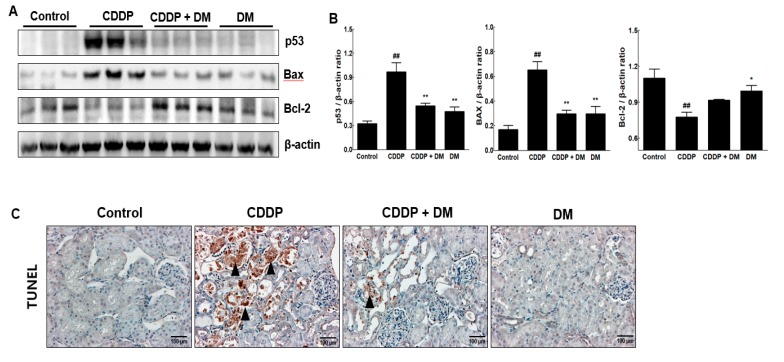
Effects of DM on apoptosis in the kidney of CDDP-treated rats. (**A**) The expression of p53, Bax, and Bcl-2 in the kidney of CDDP-treated rats was measured by Western blot analysis. β-Actin expression was used as the loading control. The Western blot results represent three separate experiments. Each group of blots is the same exposure of a gel; each experiment was repeated more than three times. (**B**) Representative graphs indicated the fold changes of Western blot data. Values are the mean ± SD of triplicate experiments. Statistical analyses were performed using one-way ANOVA followed by Tukey’s HSD post hoc test for multiple comparisons. ^##^ *p* < 0.01 compared to the control group; * *p* < 0.05 and ** *p* < 0.01 compared to the CDDP-treated group. (**C**) Detection of apoptosis via TUNEL assay in the kidney tissues of rats. Black arrowheads represent a high expression of TUNEL-positive cells. Magnification ×100.

To investigate the CDDP-induced renal damage, apoptosis-related proteins were measured in the kidney. We clearly observed that significant increases in the expression of p53 and Bax proteins levels in kidney tissue of rats treated with CDDP (Figure 7A,B). In contrast, Bcl-2 expression was significantly reduced in CDDP-treated rats. However, administration of DM markedly ameliorated the expression of CDDP-induced apoptosis-related proteins. CDDP-induced apoptosis in the kidney was also confirmed using the TUNEL assay on kidney sections. As shown in Figure 7C, a significant increase in the number of TUNEL-positive cells was found in the kidneys of rats treated with CDDP. However, DM administration dramatically reduced the number of TUNEL-positive cells, indicating that DM has antiapoptotic properties against CDDP-induced renal injury. 

### 3.7. Renoprotective Effects of DM on CDDP-Induced Tumor Xenograft Model

**Figure 8:**
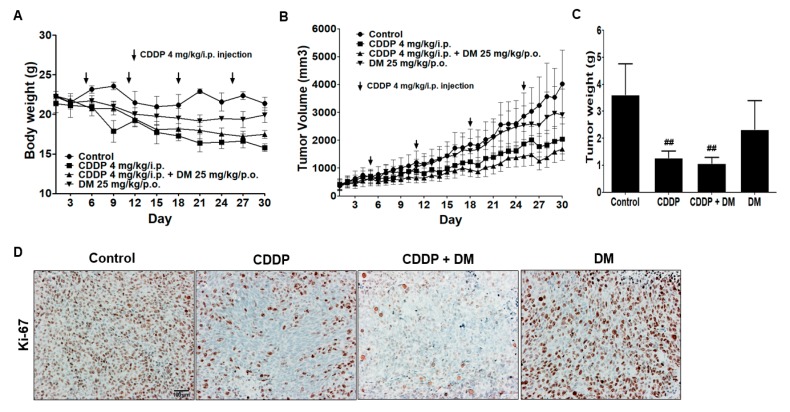
Effect of DM on CDDP-induced nephrotoxicity in tumor xenograft mice. (**A**) Effect of DM on body weight changes in tumor xenograft mice treated with CDDP. (**B**) Each graph indicates the mean tumor volumes in tumor xenograft mice after treatment with drugs for 30 days. (**C**) Each bar represents the mean tumor weight. Statistical analyses were performed using one-way ANOVA followed by Tukey’s HSD post hoc test for multiple comparisons. ^##^ *p* < 0.01 compared to the control group. (**D**) Immunohistochemical staining for Ki-67 in tumor tissues. Magnification ×100, Scale bar = 100 μm. Representative immunohistochemical images were captured under a 40× objective lens.

To evaluate the anticancer effects of CDDP in a xenograft tumor model, nude mice were inoculated with HCT-116 cells and treated with CDDP (4 mg/kg, i.p., once per week) or DM (25 mg/kg/day) for four weeks. Animals in the CDDP-treated group showed a significant decrease in body weight (Figure 8A). In CDDP-treated groups, tumor volumes and tumor weights were significantly reduced relative to the control group. Administration of DM dramatically suppressed tumor growth in a similar pattern to that observed in CDDP-treated animals (Figure 8B,C). Immunochemical staining revealed that the antitumor activity of CDDP and DM against colon tumor cell proliferation is related to a reduction in Ki-67 in tumor tissues (Figure 8D). These results indicate that combination treatment with DM and CDDP leads to a potentiation of CDDP-mediated anticancer activity. Therefore, DM did not interfere with the anticancer efficacy of CDDP in the tumor xenograft model.

**Figure 9:**
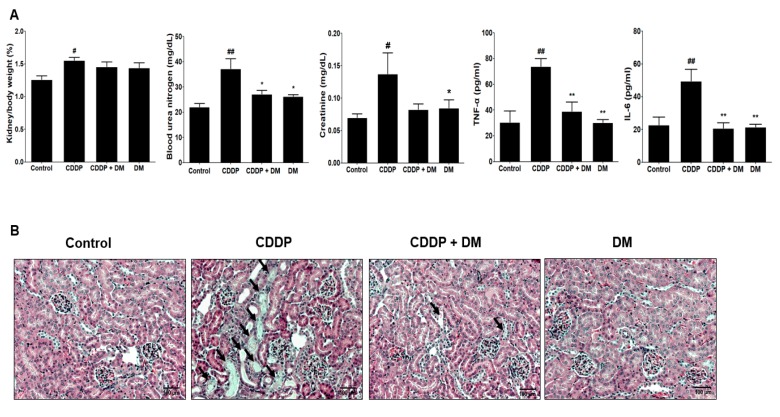
Protective effect of DM on CDDP-induced nephrotoxicity in tumor xenograft mice transfected with HCT-116 cells. (**A**) Kidney weight, nephrotoxicity biomarkers, and inflammatory cytokine levels were measured in the serum of tumor-bearing mice treated with CDDP. Values are the mean ± SD of six mice per group. Statistical analyses were performed using one-way ANOVA followed by Tukey’s HSD post hoc test for multiple comparisons. ^##^ *p* < 0.05 and ^##^ *p* < 0.01 compared to the control group; * *p* < 0.05 and ** *p* < 0.01 compared to the CDDP-treated group. (**B**) Representative histology of H&E-stained kidney sections in the experimental groups of a tumor xenograft mouse model. At 31 days, CDDP-induced acute kidney injury (AKI) mice presented an enlarged cortex with glomerular sclerosis (arrowheads). The cortex from CDDP-treated mice exhibited a normal-sized renal cortex and a lower incidence of tubular and medulla injury and displayed a normal histological structure of thin tubules and collecting ducts. Images are representative of three animals per experimental group (magnification ×100, bar = 100 μm).

In the tumor xenograft model, kidney weights were significantly increased in the CDDP-treated group compared with those in the control group. However, administration of DM reduced kidney weight to levels similar to those of the control group (Figure 9A). Therefore, these data indicate that DM protects against kidney damage induced by CDDP in tumor models. Serum BUN and sCr levels were also significantly increased in the CDDP-treated group compared with the control group (Figure 9A). These results indicate the presence of severe kidney damage in tumor-bearing mice treated with CDDP (4 mg/kg). Administration of DM significantly reduced the levels of BUN and sCr in mice treated with CDDP. No changes in BUN or sCr were observed following treatment with DM alone (Figure 9A). Similar to the results of the rat study, administration of DM significantly reduced the levels of pro-inflammatory cytokines in the tumor xenograft model by CDDP treatment (Figure 9B). As shown in Figure 9F, DM dramatically protected against CDDP-induced proximal tubular damage and disruption of renal architecture. 

**Figure 10:**
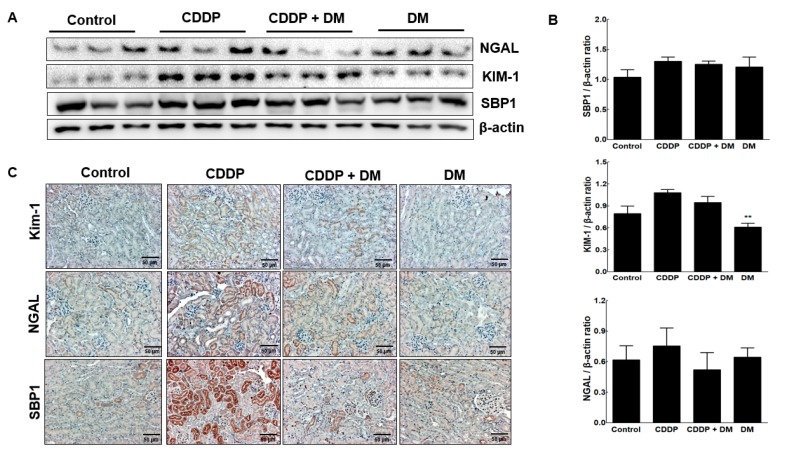
Protective effect of DM on nephrotoxicity biomarkers in CDDP-induced nephrotoxicity in tumor xenograft mice transfected with HCT-116 cells. (**A**) Nephrotoxicity biomarkers were measured in the kidney of mice. Representative Western blots of NGAL, KIM-1, and SBP1 from triplicate experiments are shown. (**B**) Densitometric analysis showed the expression of SBP1, KIM-1, and NGAL in the kidney of mice with CDDP-induced nephrotoxicity. ** *p* < 0.01 compared to the CDDP-treated group. (**C**) Representative immunohistochemical staining of KIM-1, NGAL, and SBP1 in the kidney of mice with CDDP-induced nephrotoxicity. Original magnification ×100, scale bar: 50 μm.

To assess the expression of KIM-1 and SBP1 mediated by CDDP in kidney tissues of tumor-bearing mice, protein levels of KIM-1 and SBP1 were assessed through Western blot analysis (Figure 10A). The expression levels of NGAL, KIM-1, and SBP1 were observed in the kidneys of tumor-bearing mice. As shown in Figure 10A,B, CDDP (4 mg/kg) treatment significantly increased the expression of KIM-1 and SBP1 compared with the controls. However, administration of the DM extract markedly reduced NGAL and SBP1 levels in CDDP-treated tumor-bearing mice. We also measured the expression of this biomarker via immunochemical staining (Figure 10C). NGAL, KIM-1, and SBP1 levels were mainly expressed in the damaged proximal tubules of the kidney. In particular, KIM-1 and SBP1 were highly expressed in the damaged proximal tubular area. 

## 4. Discussion

The kidney is one of the organs most vulnerable to CDDP-induced complications [22,23]. In order to prevent CDDP-induced AKI without affecting the therapeutic index, novel strategies have been investigated in cancer patients receiving CDDP chemotherapy [24,25,26]. However, no specific recommended drugs have been introduced. Recently, various drug manipulations have been proposed for protection against CDDP-induced AKI, including N-acetylcysteine, theophylline, and antioxidants. However, no treatments have been established that have no effect on the therapeutic efficacy of CDDP, and no clinical trials have demonstrated a protective effect of such treatments on CDDP-induced AKI.

*D. morbifera* exerts anti-inflammatory and antioxidant properties in various pathologies [17,18]. Nevertheless, the protective effects of the aquatic extracts from this plant against CDDP-induced AKI have not been investigated. Only a few studies have indicated the renoprotective effects of *D. morbifera* against CDDP- or cadmium-induced nephrotoxicity [27,28]. Therefore, we investigated the renoprotective effects of DM in CDDP-induced AKI through a comparison of renal injury biomarkers and antioxidant properties. To the best of our knowledge, this is the first study to demonstrate the protective effects of DM extracts against CDDP-induced AKI without affecting the anticancer therapeutic efficacy of CDDP.

In this study, exposure to a therapeutic dose of CDDP (6 mg/kg, i.p.) markedly induced AKI, as assessed by changes in the expression of renal function biomarkers such as sCr and BUN, which were significantly increased in CDDP-treated rats. Furthermore, rats with CDDP-induced AKI presented significant increases in relative kidney weight, urinary excretion of KIM-1, SBP1, NGAL, inflammatory cytokines, and fibrosis biomarkers, which are indicative of renal dysfunction. In contrast, DM administration markedly reduced these AKI biomarkers and protected against the development of AKI induced by CDDP. To investigate whether DM extract could attenuate CDDP-induced AKI, we measured sensitive biomarkers for nephrotoxicity in the urine. Similar to the changes in BUN and sCr, the levels of KIM-1, NGAL, TIMP-1, and SBP1 were markedly reduced in CDDP-treated rats following DM administration. These results are consistent with those of our previous study showing that the urinary excretion of AKI biomarkers was significantly elevated after CDDP exposure [29]. These protein-based biomarkers were observed in the urine following injury to the proximal tubule, in which the loss of reabsorption is generally indicative of injury to the tubular epithelium [30,31,32]. In this study, administration of DM protected against CDDP-induced AKI, indicating that the urinary excretion of KIM-1, NGAL, and TIMP-1 was significantly reduced in CDDP-treated rats.

Although the pathological mechanisms underlying CDDP-induced AKI are unknown, oxidative and inflammatory stress have been widely researched. Several inflammatory cytokines and chemokines are elevated in rats with CDDP-induced nephrotoxicity [33,34,35]. Our results also indicated that CDDP can induce the production of TNF-α, IL-1β, and IL-6 in the serum of rats. These results are similar to those previously reported showing that CDDP can induce the production of TNF-α, IL-1β, and IL-6 and decrease antioxidant enzyme activity [36]. However, this increase was only small compared with the control values. We also found that DM administration significantly reduced CDDP-induced increases in pro-inflammatory cytokines. The present results clearly indicate a substantial degree of oxidative stress in renal tissues of CDDP-treated mice. This was demonstrated by a significant reduction of SOD and catalase activities in the kidney tissues. CDDP affects multiple enzymes that protect cells from oxidative damage, including Cu, Zn-SOD, Mn-SOD, and catalase [37]. Decreased SOD activity, as observed in this study, could lead to incomplete scavenging of superoxide anions produced by CDDP accumulation in the kidney. Conversely, the decrease in catalase activity after CDDP administration could account for the inability of the kidney to eliminate and scavenge reactive oxygen species (ROS). Additionally, the oxidative stress observed in the current study may target multiple molecules in the cells and damage structural components in cells, such as lipids, proteins, and other organelles, with mitochondria being among the most affected.

Oxidative stress plays an important role in the pathogenesis of CDDP-induced renal injury through the production of ROS [38,39,40]. The enhanced level of oxidative stress in the kidney of CDDP-treated rats was associated with a significant increase in apoptosis. To confirm the CDDP-induced apoptosis, we measured the population of apoptotic cells using the TUNEL assay. In CDDP-treated kidney sections, a significant increase in TUNEL-positive cells was observed. Furthermore, administration of DM extract conferred protection against apoptotic cell death in the kidneys of rats in the CDDP-induced AKI group. Administration of DM extract significantly reduced the number of TUNEL-positive cells in the kidneys of rats or in tumor-bearing mice by CDDP treatment. Therefore, the present results demonstrate that CDDP induces oxidative damage in rat kidney, as evidenced by increased numbers of TUNEL-positive cells found by immunohistochemical (IHC) analysis. Oxidative stress is an important driver of CDDP-induced nephrotoxicity [41,42]. In a tumor xenograft model, we further demonstrated that DM extract significantly reduced the urinary excretion of KIM-1 and SBP1 in tumor xenograft mice by CDDP-mediated chemotherapy. Furthermore, we showed that pro-inflammatory cytokines are effective targets against renal injury in CDDP chemotherapy. Therefore, DM extract inhibits the production of pro-inflammatory cytokines and protects against CDDP-induced renal injury. The major components of DM extract are chlorogenic acid and neochlorogenic acid, which are among the most abundant polyphenol compounds in the diet. The renoprotective effect of DM extract may also be attributed to their antioxidant properties. However, DM extracts contain a large number of different types of compounds; therefore, further isolation work still needs to be undertaken to elucidate their anti-inflammatory properties. Overall, these findings suggest that DM extracts attenuate CDDP-induced AKI and that this protection may be due to their antioxidative and anti-inflammatory activities.

## 5. Conclusions

The renoprotective effect of DM against CDDP-induced nephrotoxicity was evaluated in both rat and tumor xenograft mice models. The present study highlights the potential role of DM in alleviating CDDP-induced renal dysfunction without affecting its antitumor activity by reducing ROS formation, reducing pro-inflammatory cytokine production, and inhibiting apoptosis in the kidney. Therefore, our results reveal that DM might be suitable for the protection of CDDP-induced AKI in cancer patients, without affecting chemotherapeutic efficacy.
